# Tailor-Made Pentablock Copolymer Based Formulation for Sustained Ocular Delivery of Protein Therapeutics

**DOI:** 10.1155/2014/401747

**Published:** 2014-06-22

**Authors:** Sulabh P. Patel, Ravi Vaishya, Gyan Prakash Mishra, Viral Tamboli, Dhananjay Pal, Ashim K. Mitra

**Affiliations:** Division of Pharmaceutical Sciences, School of Pharmacy, University of Missouri-Kansas City, HSB 5258, 2464 Charlotte Street, Kansas City, MO 64108, USA

## Abstract

The objective of this research article is to report the synthesis and evaluation of novel pentablock copolymers for controlled delivery of macromolecules in the treatment of posterior segment diseases. Novel biodegradable PB copolymers were synthesized by sequential ring-opening polymerization. Various ratios and molecular weights of each block (polyglycolic acid, polyethylene glycol, polylactic acid, and polycaprolactone) were selected for synthesis and to optimize release profile of FITC-BSA, IgG, and bevacizumab from nanoparticles (NPs) and thermosensitive gel. NPs were characterized for particle size, polydispersity, entrapment efficiency, and drug loading. *In vitro* release study of proteins from NPs alone and composite formulation (NPs suspended in thermosensitive gel) was performed. Composite formulations demonstrated no or negligible burst release with continuous near zero-order release in contrast to NPs alone. Hydrodynamic diameter of protein therapeutics and hydrophobicity of PB copolymer exhibited significant effect on entrapment efficiency and *in vitro* release profile. CD spectroscopy confirmed retention of structural conformation of released protein. Biological activity of released bevacizumab was confirmed by *in vitro* cell proliferation and cell migration assays. It can be concluded that novel PB polymers can serve a platform for sustained delivery of therapeutic proteins.

## 1. Introduction

Diabetic retinopathy and age-related macular degeneration (AMD) are primary vision threatening ocular diseases which affect retinal pigment epithelium (RPE), macular region of the retina, choriocapillary, and Bruch's membrane. AMD is typically observed in two forms, “wet” and “dry.” In wet AMD, choroidal neovascularization (CNV) occurs due to the leakage of blood and other fluids into the subretinal space which leads to scar formation eventually causing irreversible vision loss [1]. Many investigators reported active involvement of vascular endothelial growth factor (VEGF), a naturally occurring lipoprotein in various pathophysiological processes including AMD and diabetic retinopathy (DR) [2]. Currently, anti-VEGF antibodies such as bevacizumab and ranibizumab are indicated for the treatment of wet AMD. Bevacizumab is a full-length (149 kDa) recombinant humanized murine monoclonal antibody specific to all isoforms of VEGF [3]. Due to shorter intravitreal half-life of bevacizumab [4], current treatment requires frequent intravitreal injections to maintain therapeutic levels at retina/choroid. Frequent administrations are inconvenient and cause potential complications like retinal hemorrhage, retinal detachment, endophthalmitis, and more importantly patient noncompliance [5–7].

Various biodegradable polymeric nanoparticulate formulations have been extensively investigated for controlled delivery of protein therapeutics. Biodegradable polymers such as polycaprolactone (PCL), polylactic acid (PLA), polyglycolic acid (PGA), and polyethylene glycol (PEG) have been comprehensively studied for the preparation of protein-encapsulated nanoparticles (NPs). Recently, many investigators have applied various block copolymers such as PEG-PCL [8], PCL-PEG-PCL [9], poly lactide-co-glycolide (PLGA) [10], and PEG-PLA [11] for the development of sustained release protein formulations. However, previously published reports indicate that protein/peptide molecules suffer from rapid loss of biological activity during formulation preparation, storage, and/or release [12–15]. Acylation, with polymer degradation products (lactic acid and glycolic acid) [16, 17], accelerates hydrolysis due to lower pH caused by polymer degradation [18]. Moreover, presence of hydrophobic interfaces [19] is a potential reason for the loss of activity and/or irreversible aggregation of protein therapeutics inside the PLA, PLGA, PCL-PLA-PCL, and PLA-PEG-PLA based delivery systems. It is imperative to note that any change in protein/peptide structure, either physically or chemically, may cause immunogenicity and toxicity. A consecutive antibody response signifies safety concerns and subsequently restricts the efficacy of subsequent applications [20]. As a result, there is an urgency to develop biocompatible and biodegradable polymeric system which provides sustained release of protein therapeutics at near zero-order rate for longer periods without compromising stability and functional activity of proteins.

Therefore, the objective of this work is to synthesize and evaluate novel tailor-made PB copolymers for controlled and noninvasive delivery of protein macromolecules in the treatment of posterior segment diseases. We have synthesized novel biodegradable PB copolymers by sequential ring-opening polymerization. Various ratios and molecular weights of each block (PGA, PEG, PLA, and PCL) were selected for the synthesis to optimize sustained release profile of FITC-BSA, IgG, and bevacizumab from NPs and thermosensitive gel and combinations. Our hypothesis is that release of protein therapeutics from NPs is affected by their hydrodynamic diameters. Therefore, in this study, we have examined the effect of hydrodynamic diameter of protein molecule on entrapment efficiency and* in vitro* drug release. NPs have been characterized by particle size, polydispersity, entrapment efficiency, drug loading, and* in vitro* release profiles. Furthermore, in order to achieve continuous zero-order drug release, we have prepared and characterized a novel composite formulation comprising drug-loaded NPs suspended in thermosensitive gelling aqueous solution. Stability of released protein was also examined by CD spectroscopy and* in vitro* biological assays to ensure therapeutic efficacy of the protein.

## 2. Materials and Methods

### 2.1. Materials

Poly(ethylene glycol), (PEG: 1 kDa and 4 kDa), methoxy-PEG (550 Da), stannous octoate, *ε*-caprolactone, poly(vinyl alcohol) (PVA), and lipopolysaccharide were procured from Sigma-Aldrich (St. Louis, MO, USA). L-lactide and hexamethylenediamine (HMDI) were purchased from Acros organics (Morris Plains, NJ, USA). Micro-BCA was obtained from Fisher scientific. Mouse TNF-*α*, IL-6, and IL-1*β* ELISA kits were obtained from eBioscience Inc. Lactate dehydrogenase estimation kit and CellTiter 96 AQ_ueous_ nonradioactive cell proliferation assay (MTS) kit were obtained from Takara Bio Inc. and Promega Corp., respectively. All other reagents utilized in this study were of analytical grade.

### 2.2. Methods

#### 2.2.1. Synthesis of PB Copolymers

**Figure 1:**
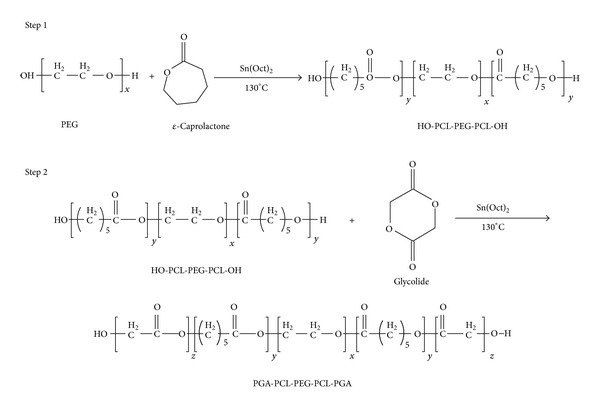
Synthesis scheme for PB-A (PGA-PCL-PEG-PCL-PGA) copolymer.

**Figure 2:**
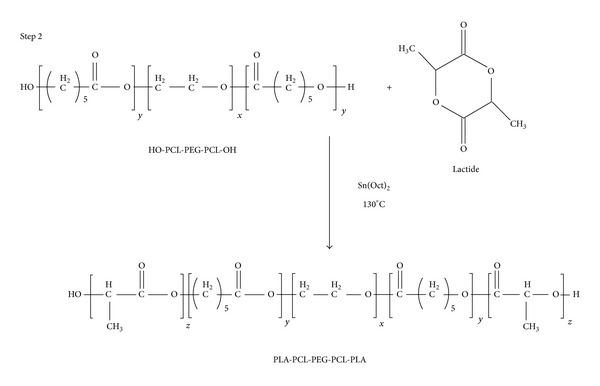
Synthesis scheme for PB-B (PLA-PCL-PEG-PCL-PLA) copolymer. Note: for synthesis of PB-B Step 1 was similar as described in Figure 1.

Novel PB copolymers, poly(glycolic acid)-poly(caprolactone)-poly(ethylene glycol)-poly(caprolactone)-poly(glycolic acid) (PGA-PCL-PEG-PCL-PGA, i.e., PB-A), poly(lactic acid)-poly(caprolactone)-poly(ethylene glycol)-poly(caprolactone)-poly(lactic acid) (PLA-PCL-PEG-PCL-PLA, i.e., PB-B), and poly(ethylene glycol)-poly(caprolactone)-poly(lactic acid)- poly(caprolactone)-poly(ethylene glycol), (PEG-PCL-PLA-PCL-PEG, i.e., PB-C), were synthesized by ring-opening bulk polymerization method [21]. PB copolymers for preparation of NPs, i.e., PB-A and PB-B, were synthesized in two steps by sequential ring-opening polymerization. PEG (1 kDa and 4 kDa) was utilized as macroinitiator and stannous octoate was utilized as catalyst. In the first step, triblock (TB) copolymer PCL-PEG-PCL (Figure 1, Step 1) was synthesized by polymerization of *ε*-caprolactone on two open hydroxyl ends of PEG. In brief, PEG (1 g, 1 kDa for PB-A) (2 g, 4 kDa for PB-B) was dissolved in anhydrous toluene followed by distillation to remove residual moisture. *ε*-Caprolactone (15 g for PB-A and 5.7 g for PB-B) and stannous octoate (0.5% w/w) were added to anhydrous PEG and temperature was raised to 130°C. After 24 h, reaction mixture was dissolved in methylene chloride followed by precipitation in cold petroleum ether. The precipitated polymer was filtered and dried for 24 h under vacuum at room temperature. In the second step, PCL-PEG-PCL TB copolymer was reacted with glycolide and L-lactide to prepare PB-A (Figure 1, Step 2) and PB-B (Figure 2) copolymers, respectively. TB copolymer and glycolide (0.6 g)/L-lactide (3.45 g) were added in round bottom flask and temperature was raised to 130°C under inert atmosphere. To this reaction mixture, stannous octoate (0.5% w/w) was added and reaction was allowed to run for 24 h. PB copolymer was then purified by cold ether precipitation method as described in the first step. The polymer was dried under vacuum and stored at −20°C until further use.

**Figure 3:**
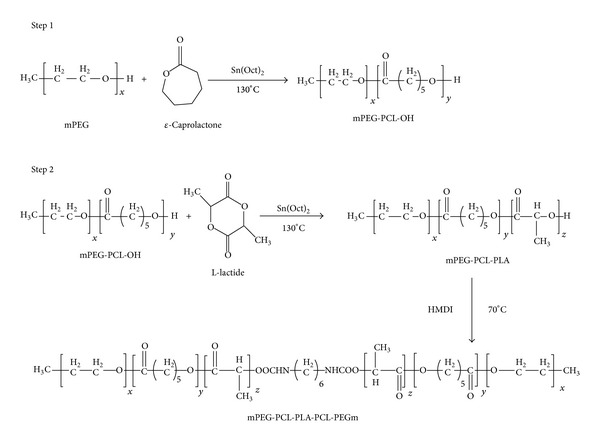
Synthesis scheme for PB-C (PEG-PCL-PLA-PCL-PEG) copolymer.

For the synthesis of thermosensitive gelling polymer, TB copolymer (mPEG-PCL-PLA) was synthesized by ring-opening bulk copolymerization as described above (Figure 3). *ε*-Caprolactone (8.25 g) was polymerized at the hydroxyl terminal of mPEG (550 Da, 5.5 g) followed by another polymerization with L-lactide (2.75 g). Resulting TB copolymers were coupled utilizing HMDI (equimolar of mPEG) as a linker. Coupling reaction was carried out at 70°C for 8 h. Polymers were purified by cold ether precipitation followed by drying under vacuum and stored at −20°C.

#### 2.2.2. Characterization of Polymers

Synthesized polymers were characterized by molecular weight and purity by ^1^H NMR spectroscopy and gel permeation chromatography (GPC).

^*1*^ *H-NMR Analysis. *To perform ^1^H-NMR spectroscopy, polymeric materials were dissolved in CDCI_3_ and spectra were recorded with Varian-400 NMR instrument. Purity and molecular weight (Mn) were calculated from the ^1^H-NMR spectra.

*GPC Analysis. *Purity, molecular weights, and polydispersity of PB copolymers were further confirmed by GPC analysis. Polymeric samples were analyzed with refractive index detector (Waters 410). Briefly, samples were prepared by dissolving 5 mg of polymeric material in tetrahydrofuran (THF) whereas THF was utilized as eluting agent at the flow rate of 1 mL/min. Separation was carried out on Styragel HR-3 column. Polystyrene samples with narrow molecular weight distribution were considered as standards.

#### 2.2.3. *In Vitro* Cytotoxicity Studies

*Cell Culture. *Human retinal pigment epithelial cells (ARPE-19) were cultured and maintained according to the previously published protocol from our laboratory [22]. In brief, cells were cultured in Dulbecco's Modified Eagle's Medium (DMEM)/F-12 medium containing 10% fetal bovine serum (FBS), 15 mM of HEPES, 29 mM of sodium bicarbonate, 100 U/L of penicillin, and 100 mg/L of streptomycin. A mouse macrophage cell line, RAW-264.7, was cultured and maintained in DMEM supplemented with 10% FBS, 100 U/L of penicillin, and 100 mg/L of streptomycin. The Statens Seruminstitut rabbit corneal (SIRC) cell line was selected between passages 410 and 425. This cell line was grown with cell culture media composed of MEM containing 10% FBS, lactalbumin, HEPES, sodium bicarbonate, 100 U/L of penicillin, and 100 mg/L of streptomycin. Human conjunctival epithelial cells (HCEC) were maintained in cell culture flask containing MEM Earle's BSS medium supplemented by 10% FBS, 100 U/L of penicillin, 100 mg/L of streptomycin, 29 mM of sodium bicarbonate, and 2 mM L-glutamine. Choroid-retinal endothelial cells (RF/6A cells) were cultured and maintained in cell culture medium composed of RPMI-1640 comprising 10% FBS, 100 U/L of penicillin, and 100 mg/L of streptomycin [23]. All five cell lines were procured from ATCC and maintained according to ATCC guideline at 37°C, 5% CO_2_, and 95% humidity.

*Lactate Dehydrogenase (LDH) Assay*. Previously published protocol with minor modification was employed to evaluate the cytotoxicity of PB copolymers [24]. Briefly, 10 mg/mL of PB copolymers (PB-A and PB-B) was dissolved in ACN and 100 *μ*L of solutions was aliquoted in each of the 96-well plates. Plates were exposed overnight under UV light (laminar flow) for polymer sterilization as well as evaporation of ACN. ARPE-19 cells at a density of 1.0 × 10^4^ were seeded in each well and incubated at 37°C and 5% CO_2_ in humidified atmosphere for 48 h. After completion of incubation period, cell supernatants were analyzed for quantification of LDH. Absorbance of each well was estimated at 450 nm by 96-well plate reader. More than 10% of LDH release was considered as cytotoxic. To evaluate cytotoxicity of block copolymers on conjunctiva, cornea, and macrophages, similar experiment was performed with HCEC, SIRC, and RAW-264.7 cells. LDH release (%) was calculated according to

(1) LDH  release(%)=Abs.  of  Sample−Abs.  of  negative  controlAbs.  of  positive  control−Abs.  of  negative  control ∗100.

*MTS Assay. *Safety and biocompatibility of PB copolymers were further established by performing* in vitro* cell viability assay (MTS assay) [25]. This assay was performed according to previously reported protocol with minor modifications. As described earlier, PB copolymers solutions at the concentration of 10 mg/mL were prepared, aliquoted, and sterilized. After sterilization, ARPE-19 cells were seeded in each well of 96-well plates at a cell density of 1.0 × 10^4^ and incubated at 37°C and 5% CO_2_ in humidified atmosphere for 48 h. At the end of incubation period, cell culture medium was aspirated and cells were incubated for 4 h (37°C and 5% CO_2_) in presence of 100 *μ*L of serum-free medium containing 20 *μ*L MTS solution. Absorbance of each well was estimated at 450 nm. Similar procedure was repeated with other ocular and macrophage cell lines such as HCEC, SIRC, and RAW-264.7 cells. Percent cell viability was calculated according to

(2) Cell  viability(%)=Abs.  of  Sample−Abs.  of  negative  controlAbs.  of  positive  control−Abs.  of  negative  control ∗100.

In this study, PB copolymers which exhibited more than 90% of cell viability were considered nontoxic and suitable for ocular applications.

#### 2.2.4. *In Vitro* Biocompatibility Studies

PB copolymers were dissolved in ACN at the concentration of 10 mg/mL. Two hundred *μ*L was aliquoted in each well of 48-well cell culture plates which were incubated overnight under UV lights (laminar flow) for ACN evaporation and sterilization of resulting polymer film. After sterilization, RAW-264.7 cells (5.0 × 10^4^) were seeded in each well of cell culture plate and incubated for 24 h at 37°C and 5% CO_2_. Cell supernatants were analyzed for the presence of cytokines, that is, TNF-*α*, IL-6, and IL-1*β*. Lipopolysaccharide (LPS) was utilized as positive control whereas cells without treatment were considered as negative control. Cytokines were measured by ELISA method according to manufacturer's instructions. Standard calibration curves for TNF-*α*, IL-6, and IL-1*β* were prepared in ranges of 10–750 pg/mL, 5–500 pg/mL, and 10–500 pg/mL, respectively.

#### 2.2.5. Preparation of Nanoparticles

IgG-loaded PB NPs were prepared by W_1_/O/W_2_ double emulsion solvent evaporation method [26]. Briefly, predetermined quantity of IgG (10 mg) was dissolved in 0.1 M phosphate buffer saline (PBS, pH 7.4) (1 mL) containing 50 *μ*L of Tween-80 (W_1_ phase). In order to prepare organic phase, 100 mg of PB copolymer was solubilized in 4 mL of dichloromethane (DCM) containing 50 *μ*L of Span-20 (organic phase). Primary emulsion (W_1_/O) was prepared by dropwise addition of W_1_ phase to the organic phase under constant sonication applied with probe-sonicator for 1 min at 4 W output. To avoid excessive heating and possible degradation of protein, preparation of emulsion was carried out in ice-bath. Resulting W_1_/O primary emulsion was then added dropwise in 20 mL of 2% polyvinyl alcohol (PVA) solution (W_2_ phase) under constant sonication for 4 min at 5 W output. Double emulsion (W_1_/O/W_2_) was stirred at room temperature for 30 min followed by evaporation of DCM under low pressure. Once DCM was evaporated, NPs were centrifuged for 30 min at 20000 rpm and 4°C followed by two washing cycles with distilled deionized water (DDW). Finally, IgG-loaded NPs were freeze-dried in presence of 5% mannitol (cryoprotectant) and stored at −20°C until further use. NPs were prepared with two PB copolymers, that is, PB-A and PB-B. A similar protocol was followed to prepare FITC-BSA and bevacizumab-loaded NPs. NPs were characterized by particle size, entrapment efficiency (EE%), drug loading (DL%), and* in vitro* drug release pattern.

#### 2.2.6. Characterization of Nanoparticles

*Particle Size and Polydispersity. *Freeze-dried NPs were dispersed in DDW (1 mg/mL) and analyzed for their size and distribution. Particle size was determined by Zeta sizer (Zetasizer Nano ZS, Malvern Instruments Ltd., Worcestershire, UK) at 90° scattering angel. All the NP samples were analyzed in triplicate.

*Entrapment Efficiency (EE) and Drug Loading (DL). *Protein-encapsulated freeze-dried NPs were evaluated for the estimation of entrapment efficiency (EE) and drug loading (DL). EE was estimated by the amount of protein in the supernatants obtained from NP preparation. Micro-BCA protein estimation kit was employed for estimation of total protein. For analysis of DL, 2 mg equivalent protein-loaded NPs was dissolved in 200 *μ*L of dimethyl sulfoxide (DMSO). Resulting solutions were analyzed by UV absorbance spectroscopy. Standard curve of respective proteins (IgG, FITC-BSA, and bevacizumab) ranging from 31.25 to 2000 *μ*g/mL was prepared in DMSO. Following equations were utilized for the calculation of EE (%) and DL (%):

(3) EE  (%)=(1−Amount  of  drug  in  supernatantTotal  amount  of  drug)∗100,DL  (%)=(Amount  of  drug  in  nanoparticlesTotal  amount  of  drug  and  polymer)∗100.

*In Vitro Release Studies. *IgG-loaded NPs were further characterized by their ability to sustain drug release. In order to conduct* in vitro* drug release studies, 1 mg of IgG equivalent freeze-dried NPs was suspended in 1 mL of PBS (pH 7.4). Resulting NP suspension was then incubated in water bath equilibrated at 37°C. At predefined time intervals, the suspension was centrifuged at 13000 rpm for 30 min. A 200 *μ*L supernatant was collected and replaced with the same volume of PBS. NPs were then resuspended and release study was continued at 37°C. In a second set of* in vitro* release studies, 1 mg equivalent IgG containing NPs was suspended in 500 *μ*L of aqueous solution of thermosensitive gelling polymer (PB-C) (20 wt%). Resulting suspension was incubated in 10 mL vial at 7°C for 30 min. Once gel was formed, an aliquot (5 mL) of PBS (preincubated at 37°C) was slowly added. At predetermined time intervals, 1 mL of clear supernatant was collected and replaced with the same volume of fresh PBS (preincubated at 37°C). Release samples were analyzed by Micro-BCA for total protein content according to supplier's instructions. FITC-BSA and bevacizumab-loaded NPs were also evaluated for* in vitro* release kinetics. Samples of released FITC-BSA were analyzed by fluorescence spectroscopy with excitation and emission wavelengths of 490 nm and 525 nm, respectively.* In vitro* release experiments were performed in triplicate and expressed as cumulative drug released (%) with time.

#### 2.2.7. Release Kinetics

In order to delineate release mechanism, release data was fitted to five different kinetic models described below.

Korsmeyer-Peppas equation is as follows:

(4) $$\begin{array}{c} \frac{M_{t}}{M_{\infty }}=\;kt^{n}, \end{array}$$

where *k* is the kinetic constant and *n* is the diffusion exponent which describes release mechanism. *M*_*t*_ and *M*_*∞*_ represent cumulative protein release at time *t* and at the equilibrium, respectively.

Higuchi equation is as follows:

(5) $$\begin{array}{c} Q_{t}=Kt^{1/2}, \end{array}$$

where *K* denotes Higuchi rate kinetic constant, *Q*_*t*_ is the amount of released protein at time *t*, and *t* is time in hour.

Hixson-Crowell equation is as follows:

(6) $$\begin{array}{c} {C_{0}}^{1/3}-{C_{t}}^{1/3}=kt, \end{array}$$

where *C*_0_ and *C*_*t*_ represent initial and remaining amounts of protein in formulation, respectively, *k* is the constant incorporating surface to volume ratio, and *t* is time in hour.

First-order equation is as follows:

(7) $$\begin{array}{c} \log C=\text{Log}C_{0}-\frac{Kt}{2.303}, \end{array}$$

where *K* denotes the first-order rate constant, *C*_0_ is the initial protein concentration, and *t* represents time in hour.

Zero-order equation is as follows:

(8) $$\begin{array}{c} C=K_{0}t, \end{array}$$

where *K*_0_ is the zero-order rate constant and *t* is time in hour.

#### 2.2.8. Stability of IgG

Released IgG was analyzed by CD spectroscopy to examine secondary structure. CD analysis was carried out at room temperature with Jasco 720 spectropolarimeter. CD spectra were recorded between wavelengths of 200 and 250 nm at scanning speed of 5 nm/min utilizing 1 cm cell. CD measurements were reported as molar ellipticity [*θ*]. CD spectrum of PBS was utilized as blank.

#### 2.2.9. Stability Study of Bevacizumab by* In Vitro* Biological Assays

*Cell Proliferation Assay. *A cell proliferation assay (MTS) was performed according to previously published protocols with minor modifications [27–29]. Briefly, cells were seeded at a density of 5 × 10^3^ cells/well of 96-well cell culture plates. After 24 h of incubation, cells were serum-starved overnight followed by addition of serum-free medium containing 100 ng/mL of VEGF and 0.25 mg/mL of released bevacizumab (test samples) or 0.25 mg/mL of native bevacizumab (standard). Cells without VEGF or with native bevacizumab treatment were considered as negative control or cells exposed only to VEGF (100 ng/mL) as positive control. Incubation was carried out for 24 h at 37°C and 5% CO_2_. Next, cells were exposed to 100 *μ*L of serum-free medium containing 20 *μ*L of MTS solution and incubated for 4 h. The absorbance was recorded at 450 nm using a microplate reader.

*Cell Migration Assay. *A cell migration assay was performed as described elsewhere [30] with few modifications. RF/6A cells were starved overnight (by exposing it to serum-free medium), trypsinized, and suspended in serum-free medium containing 0.25 mg/mL of bevacizumab (native or released from NPs). Cells (5 × 10^3^) were seeded in upper chamber of Transwell (8.0 *μ*m pore size, 10 mm diameter, Corning Inc.) and preincubated with cell culture medium. VEGF (100 ng/mL) was placed into the lower chamber and cells were incubated for 24 h at 37°C and 5% CO_2_. Nonmigrated cells were removed from upper chamber by cotton swab and concentration of migrated cells was estimated by AlamarBlue assay. It was performed according to supplier's protocol. Moreover, for visual evidence, migrated cells were also stained with methylene blue and images were taken with Leica DMI3000B inverted microscope (Germany). Each experiment was repeated three times.

## 3. Results and Discussion

### 3.1. Synthesis and Characterization of PB Copolymers

**Figure 4:**
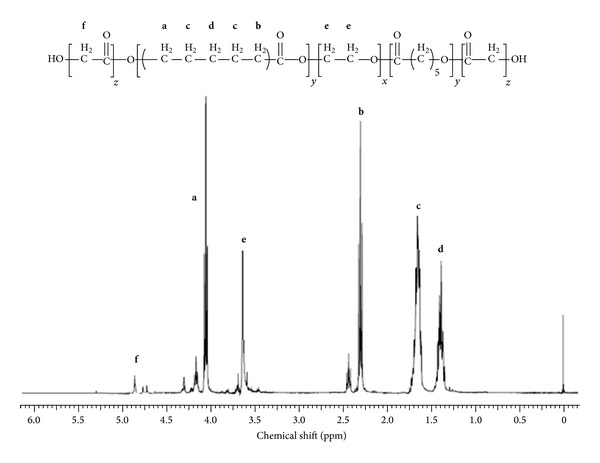
^1^H-NMR spectra of PB-A copolymer in CDCl_3_.

**Figure 5:**
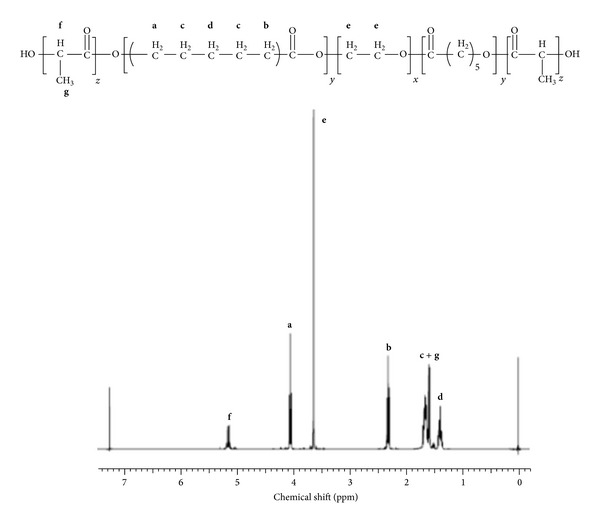
^1^H-NMR spectra of PB-B copolymer in CDCl_3_.

**Figure 6:**
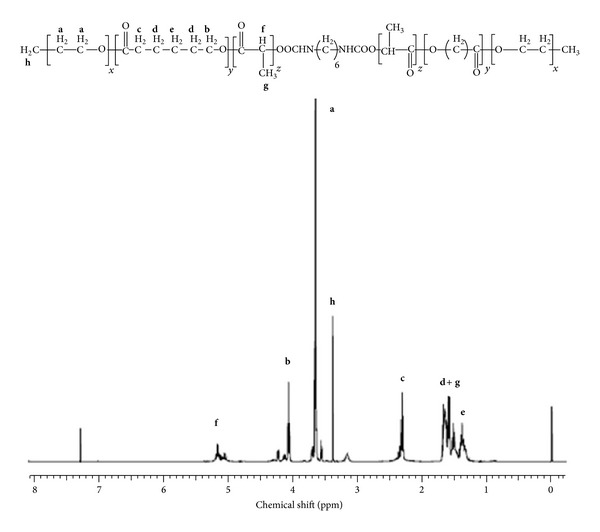
^1^H-NMR spectra of PB-C copolymer in CDCl_3_.

**Figure 7:**
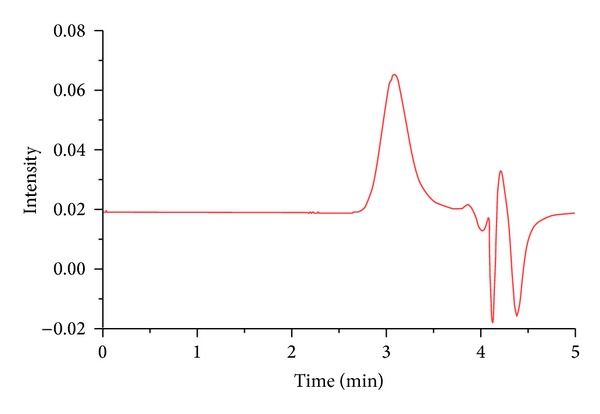
Gel permeation chromatogram of PB-A copolymer.

PB copolymers (PB-A and PB-B) were successfully synthesized by ring-opening bulk copolymerization of *ε*-caprolactone and L-lactide/glycolide. In the first step, TB copolymers (PCL-PEG-PCL) were synthesized, purified, and characterized as mentioned in the method section. Purified TB copolymers were then utilized for the synthesis of respective PB copolymers, that is, PB-A (PGA-PCL-PEG-PCL-PGA) and PB-B (PLA-PCL-PEG-PCL-PLA). Purity and molecular weights (Mn) of PB copolymers were calculated by ^1^H-NMR spectroscopy. As described in Figures 4 and 5, typical ^1^H-NMR signals of PCL blocks were observed at 1.40, 1.65, 2.30, and 4.06 ppm depicting methylene protons of –(CH_2_)_3_–, –OCO–CH_2_–, and –CH_2_OOC–, respectively. PB-A exhibited cluster of singlets between 4.6 and 4.9 ppm representing methylene protons (–CH_2_–) of PGA units. PB copolymer with PLA units as terminals (PB-B) demonstrated two additional peaks at 1.50 (–CH_3_) and 5.17 (–CH–) ppm. Molar ratios of PB-A and PB-B were calculated from the integration values of PEG (3.65 ppm), PCL (2.30 ppm), and PLA (5.17 ppm) or PGA (4.6–4.9 ppm). ^1^H-NMR spectra of PB-C (Figure 6) exhibit typical proton signals of PEG, PCL, and PLA. An additional peak at 3.38 ppm was denoted to terminal methyl of (–OCH_3_–) of PEG and utilized for the molecular weight (Mn) calculation of PB-C copolymer. As described in Table 1, polydispersity (PDI) of all the polymers was below 1.45 suggesting narrow distribution of molecular weights. Moreover, block copolymers depicted a single peak in GPC chromatogram (Figure 7) indicating monodistribution of molecular weight and absence of any homopolymers such as PLA, PGA, PCL, and PEG. Molecular weights (Mn) of PB-A, PB-B, and PB-C calculated with ^1^H-NMR spectroscopy and GPC were very similar to theoretical molecular weights (Table 1). Therefore, theoretical molecular weights are mentioned instead of calculated molecular weights in this report.

### 3.2. *In Vitro* Cytotoxicity Studies

**Figure 8:**
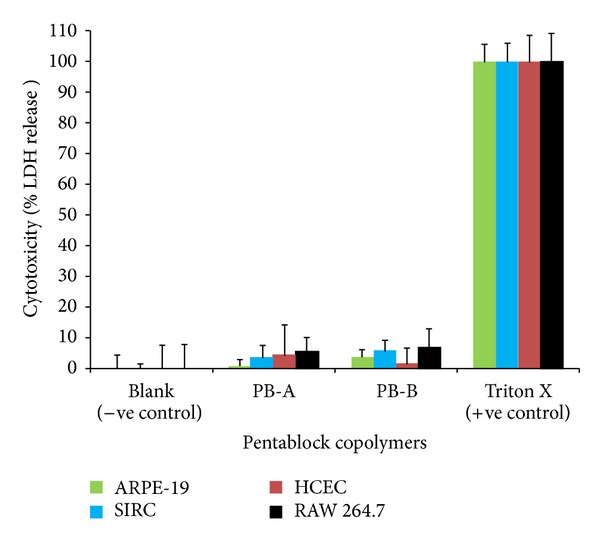
*In vitro* cytotoxicity assay (LDH) of PB-A and PB-B copolymers at the concentration of 10 mg/mL was performed on ARPE-19, SIRC, HCEC, and RAW-264.7 cell lines. Results are described in mean ± SD, *n* = 6.

In order to investigate compatibility of PB polymeric materials with biological system (ocular cell lines), ARPE-19, SIRC, HCEC, and RAW-264.7 cells for 48 h were treated with 10 mg/mL of PB-A and PB-B for 48 h. LDH is a cytoplasmic enzyme, secreted in cell culture medium following cell-membrane damage. Estimation of LDH concentration in culture supernatant may provide PB copolymer toxicity. Less than 10% of LDH release was observed after 48 h exposure indicating negligible toxicity with any of the ocular cell lines (Figure 8). Noticeably, results were comparable with negative controls.

**Figure 9:**
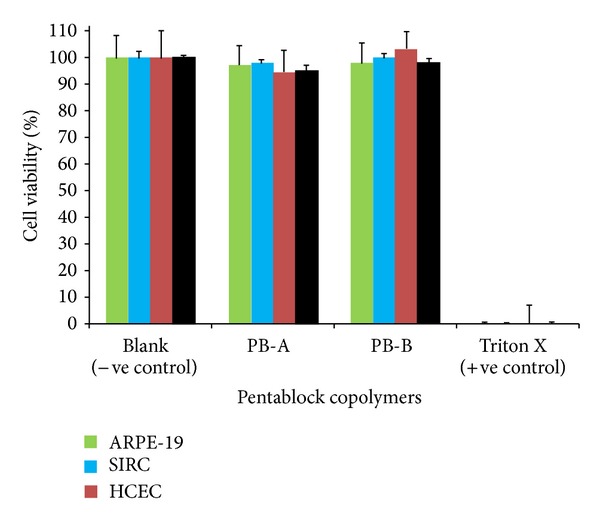
*In vitro* cell viability assay (MTS) of PB-A and PB-B copolymers at the concentration of 10 mg/mL was performed on ARPE-19, SIRC, HCEC, and RAW-264.7 cell lines. Results are described in mean ± SD, *n* = 6.

To further confirm our observation, in addition to LDH assay, MTS cell viability studies were also performed utilizing a similar protocol. In MTS assay, only metabolically active cells convert tetrazolium compound to formazan. Hence, the concentrations of formazan products provide a direct estimation of cell viability. Results in Figure 9 demonstrate that more than 90% cell viability (for all the cell lines) after 48 h exposure to polymeric materials suggesting excellent safety profile of block copolymers for ocular applications. No significant difference in cell viability was observed relative to negative control.

### 3.3. *In Vitro* Biocompatibility Studies

**Figure 10:**
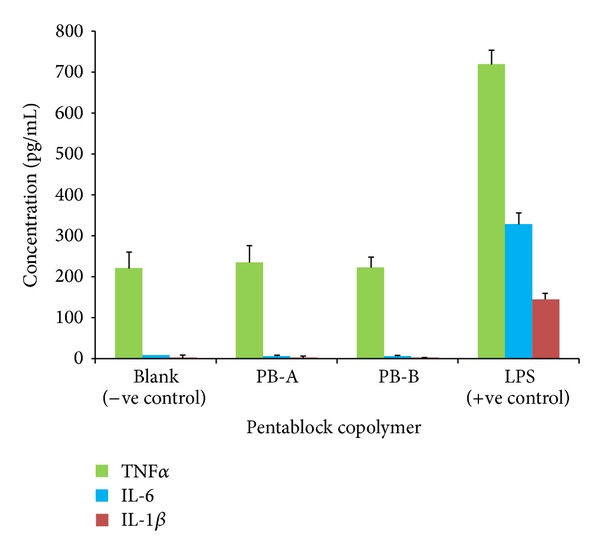
*In vitro* biocompatibility of PB-A and PB-B copolymers was evaluated by estimating the levels of TNF-*α*, IL-6, and IL-1*β* in the supernatants of polymer treated RAW 264.7 cells. Results are described in mean ± SD, *n* = 6.

Many investigators have utilized* in vitro* cell culture model (RAW-264.7) for the estimation of biocompatibility of polymeric materials intended for human applications. In this study we have examined various cytokines release such as TNF-*α*, IL-6, and IL-1*β* in culture supernatant following 24 h exposure to PB-A and PB-B copolymers. Samples were analyzed via sandwich ELISA method. Results depicted in Figure 10 indicate release of TNF-*α* (~200 pg/mL) in both groups, that is, PB-A and PB-B. However, these values are comparable to negative control (cells without treatment) with no significant difference. Similarly, negligible release of IL-6 and IL-1*β* was observed suggesting that these copolymers are safe for human use.

### 3.4. Characterization of NPs

#### 3.4.1. Particle Size and Polydispersity

IgG, FITC-BSA, and bevacizumab-encapsulated PB NPs were prepared by W_1_/O/W_2_ double emulsion solvent evaporation method. NPs prepared from PB copolymers were ranging in diameter from 320 to 355 nm (Table 2). Unimodal size distribution with very narrow polydispersity (0.273–0.305) was noted. No significant effect of polymer composition (PB-A/PB-B) or type of protein molecule (IgG/BSA/bevacizumab) was evident on particle size. These results suggest that hydrodynamic diameter of protein therapeutics or polymer composition has no significant effect on particle size or distribution.

#### 3.4.2. Entrapment Efficiency (EE) and Drug Loading (DL)

Entrapment efficiency (EE) and drug loading (DL) are highly influenced by various parameters including copolymer composition (hydrophobicity of polymer) and phase volume ratios (W_1_, O, and W_2_). In order to understand the effect of hydrophobicity of copolymers on EE and DL, we have prepared and evaluated IgG and FITC-BSA-loaded NPs utilizing PB-A and PB-B. PB-A copolymer comprising PGA (hydrophilic block) is relatively hydrophilic compared to PB-B copolymer comprising PLA (hydrophobic block). As presented in Table 2, encapsulation of IgG or FITC-BSA in PB-A NPs was ~40% and ~35%, respectively. However, PB-B NPs exhibited significantly higher encapsulation of ~70% and ~69% for IgG and FITC-BSA, respectively. This is possibly due to the fact that, during preparation of NPs (solvent evaporation), hydrophobicity may allow faster precipitation of PB-B copolymer to form NPs preventing diffusion of IgG/FITC-BSA from W_1_ phase to external aqueous (W_2_) phase. This phenomenon ensured higher EE of protein therapeutics in PB-B NPs. Due to higher hydrophilicity, PB-A copolymer may remain hydrated with W_1_ and W_2_ phases (during NPs preparation) allowing escape of IgG/FITC-BSA in external phase resulting in poor EE. However, no effect of hydrodynamic diameter of IgG or FITC-BSA on EE or DL was observed. It is very plausible that both of the proteins are too large (≥66 kDa) to show any significant effect of hydrodynamic diameter on EE or DL. Bevacizumab-loaded PB-B NPs exhibited ~67% of EE and ~6% of DL, very similar to IgG-loaded PB-B NPs (Table 2) suggesting that similar molecules behave similarly during NP preparation.

#### 3.4.3. * In Vitro* Release Studies

**Figure 11:**
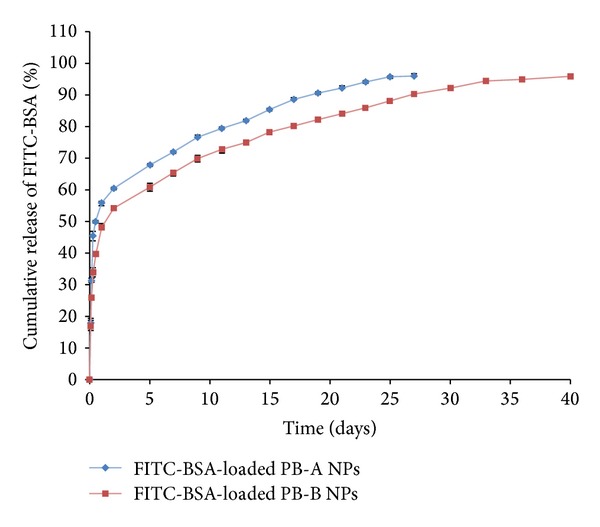
*In vitro* release of FITC-BSA from NPs prepared with PB-A and PB-B copolymers. Results are described in mean ± SD, *n* = 3.

**Figure 12:**
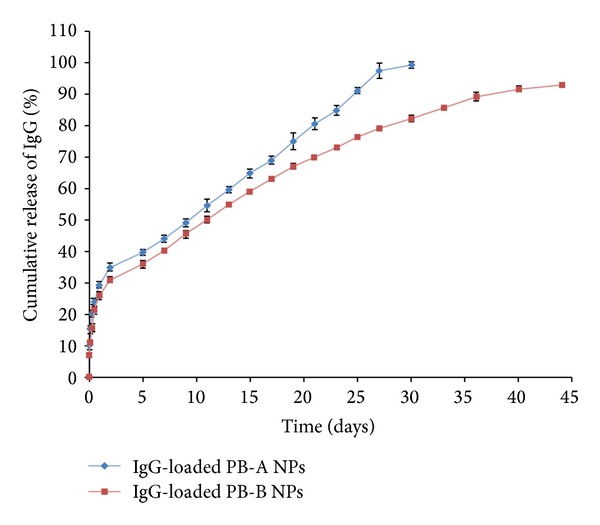
*In vitro* release of IgG from NPs prepared with PB-A and PB-B copolymers. Results are described in mean ± SD, *n* = 3.

In order to evaluate the effect of polymer hydrophobicity, release of FITC-BSA and IgG from PB-A and PB-B NPs was evaluated. As described in Figure 11, both NPs (PB-A and PB-B) demonstrated biphasic release profile, that is, initial burst release followed by sustained release. PB-A NPs exhibited significantly higher burst release (~56%) of FITC-BSA relative to PB-B NPs (~48%). In the second phase, PB-B NPs provided sustained BSA release for ~36 days whereas PB-A NPs prolonged the release for only ~27 days. Similar effect of polymer hydrophobicity was observed with IgG-encapsulated NPs where PB-B NPs displayed longer release (~44 days) than PB-A NPs (~30 days) (Figure 12). As described earlier, PGA based PB-A copolymer is hydrophilic compared to PLA based PB-B copolymer. Therefore, it is anticipated that PB-A NPs possibly have higher affinity for the protein molecules which may result in higher amount of surface adsorbed drug. Moreover, being hydrophilic, PB-A NPs may be easily hydrated which may allow rapid diffusion of water molecules through polymeric matrix. Therefore, higher amounts of surface adsorbed proteins and higher affinity towards water molecules may have contributed to the higher burst release and shorter duration of release from in PB-A NPs.

**Figure 13:**
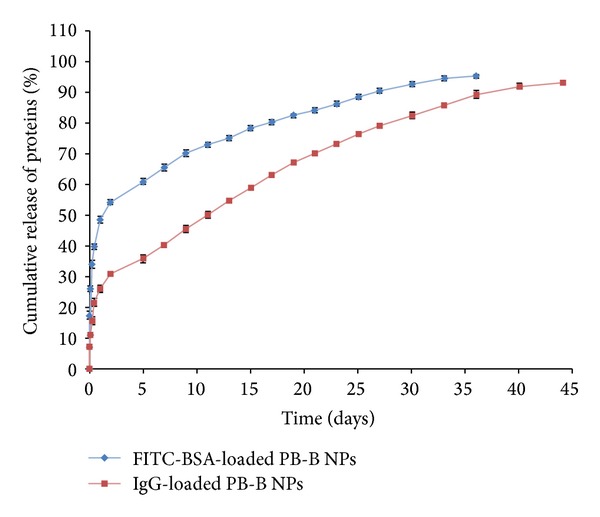
*In vitro* release of FITC-BSA and IgG from PB-B NPs. Results are described in mean ± SD, *n* = 3.

**Figure 14:**
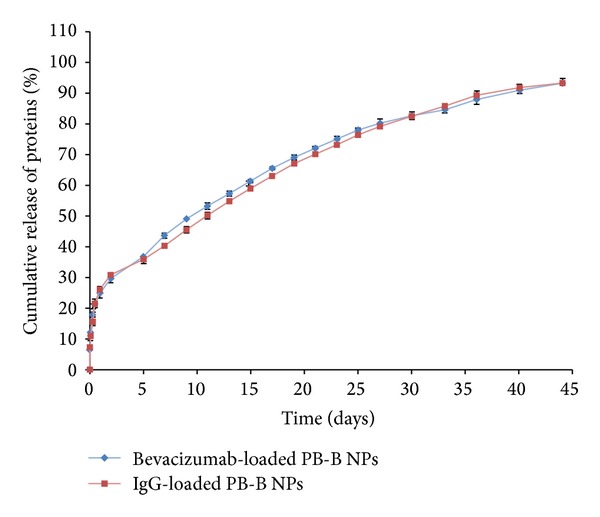
*In vitro* release of IgG and bevacizumab from PB-B NPs. Results are described in mean ± SD, *n* = 3.

Hydrodynamic diameter of protein therapeutics may have significant effect on drug release pattern. In order to examine this possibility we have compared* in vitro* release profiles of FITC-BSA (66 kDa) and IgG (150 kDa) (Figure 13) from PB-B NPs. Results indicate significantly higher burst release and shorter release duration for FITC-BSA relative to IgG from their respective NPs. FITC-BSA has smaller hydrodynamic diameter compared to IgG and may lead to more rapid diffusion through the polymeric matrix of NPs. To further confirm this hypothesis, we have compared* in vitro* release profile of IgG and bevacizumab (149 kDa) from their respective PB-B NPs.  Figure 14 suggests no significant difference between the release profile of IgG and bevacizumab. Since IgG and bevacizumab are full-length antibodies with similar hydrophilicity and molecular weight (hydrodynamic diameter), both protein molecules behave similarly during NPs preparation and during release. These results suggest that parallel molecules (IgG or bevacizumab) may not generate any difference in drug release pattern whereas there is a significant effect of hydrodynamic diameter (FITC-BSA or IgG) on drug release.

Protein molecules exhibit high specificity and require very low dose to exert therapeutic activity. Any nanoparticulate or microparticulate system can have certain amount of surface adsorbed drug, which is released within the first 24 h leading to burst effect. Being very potent by nature, burst release of protein therapeutics may produce side effects. Therefore, pharmaceutical scientists are focused on developing a formulation which can eliminate or minimize burst effect and offer zero-order drug release throughout the release period. In order to achieve zero-order drug release profile, protein-encapsulated PB-B NPs were suspended in an aqueous solution of thermosensitive gelling polymer (PB-C). Thermosensitive gelling solution was composed of 20 wt% PB-C copolymer in DDW. Aqueous solution of PB-C copolymer remains in liquid state around room temperature but immediately transforms to hydrogel at body temperature (sol-gel transition).

**Figure 15:**
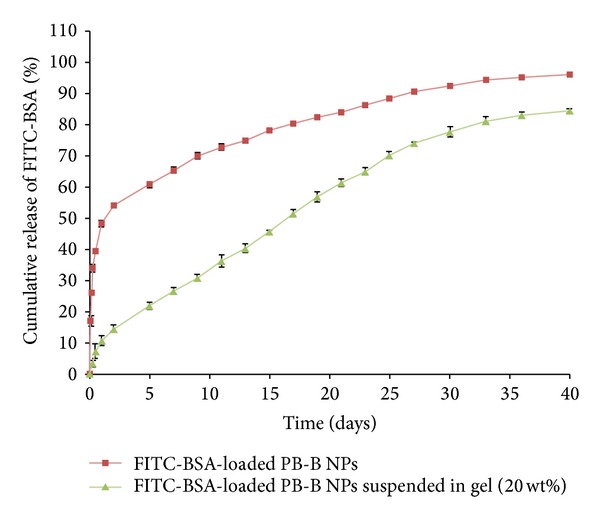
*In vitro* release of FITC-BSA from PB-B NPs and PB-B NPs suspended in PB-C gelling polymer. Results are described in mean ± SD, *n* = 3.

**Figure 16:**
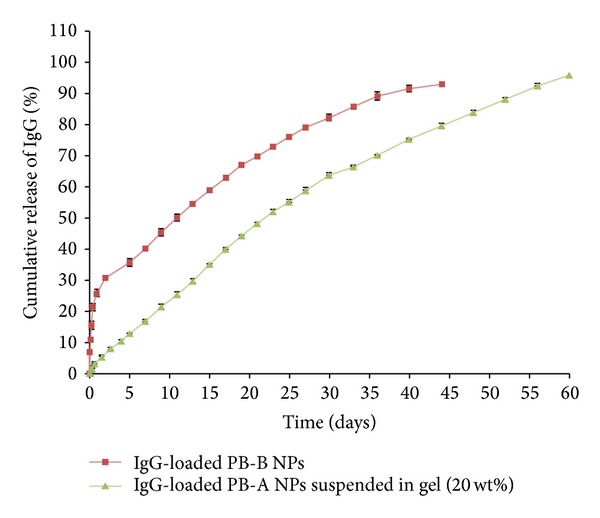
*In vitro* release of IgG from PB-B NPs and PB-B NPs suspended in PB-C gelling polymer. Results are described in mean ± SD, *n* = 3.

Protein-loaded NPs were suspended in 20 wt% gelling solution and then brought to 37°C which immediately transitioned the solution to solid hydrogel entrapping NPs throughout the polymeric matrix. These composite formulations comprised of protein-loaded PB-B NPs (FITC-BSA and IgG) suspended in thermosensitive gel were evaluated for release pattern. As depicted in Figure 15, burst release of FITC-BSA from composite formulation was negligible (~10%) relative to burst release observed from PB-B NPs (~48%). In addition, release of FITC-BSA was prolonged over 40 days. A similar pattern was also observed for a composite formulation comprising IgG-loaded PB-B NPs suspended in 20 wt% thermosensitive gelling solution (Figure 16). Composite formulation of IgG exhibited negligible burst release followed by zero-order release up to 60 days. This behavior may be due to the fact that when the NPs were suspended into the gel matrix the matrix serves as an additional diffusion barrier for the surface adsorbed drug. The gel matrix might deter quick hydration and dumping of surface adsorbed dose eliminating burst effect. It also leads to zero-order drug release throughout the release period.

### 3.5. Release Kinetics

In order to evaluate drug release mechanism, we have fitted* in vitro* drug release data in five different release kinetic models, that is, Korsmeyer-Peppas, Higuchi, Hixson-Crowell, zero-order, and first-order models. Results, presented in Table 3, indicate that Korsmeyer-Peppas is the best fit model for all the formulations with *R* ^2^ values ranging between 0.977 and 0.997. Moreover, *n* values in Korsmeyer-Peppas model for release of FITC-BSA, IgG, and bevacizumab from PB-B NPs were below 0.43 indicating diffusion controlled release. Interestingly, *n* values for composite formulation of FITC-BSA (0.549) and IgG (0.818) (NPs suspended in thermosensitive gel) were between 0.43 and 0.89 suggesting anomalous diffusion. In other words, release of protein therapeutics from composite formulation is controlled by diffusion as well as degradation of polymer.

### 3.6. Stability of Secondary Structure of IgG

**Figure 17:**
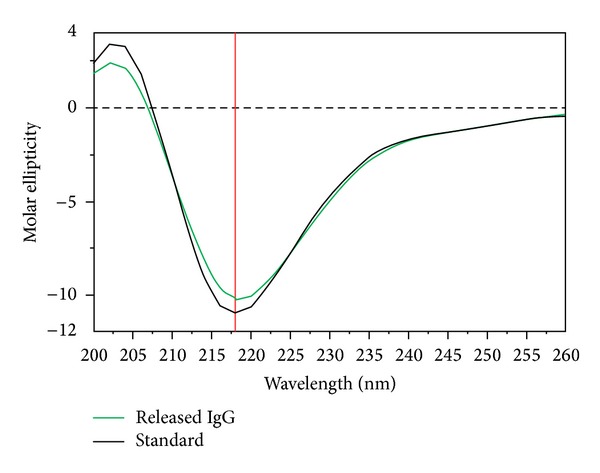
Stability of released IgG confirmed by CD spectroscopy.

Protein molecules need to maintain three-dimensional structure to retain biological activity. CD spectroscopy is a sensitive and robust analytical technique exploited for the investigation of secondary and to some extent tertiary conformation of proteins. It is sensitive enough to detect minor conformational changes in *α*-helix and *β*-sheets of the protein. Therefore, we have utilized this technique to confirm the conformational stability of released IgG by comparing with CD spectrum of native IgG (Figure 17). CD spectrum of released IgG demonstrated *λ* minima of 218 nm similar to the native IgG. In addition, CD spectra of native and released IgG ranging from 200 nm to 250 nm were identical indicating retention of protein conformation during NP preparation and after release.

### 3.7. Cell Proliferation Assay

**Figure 18:**
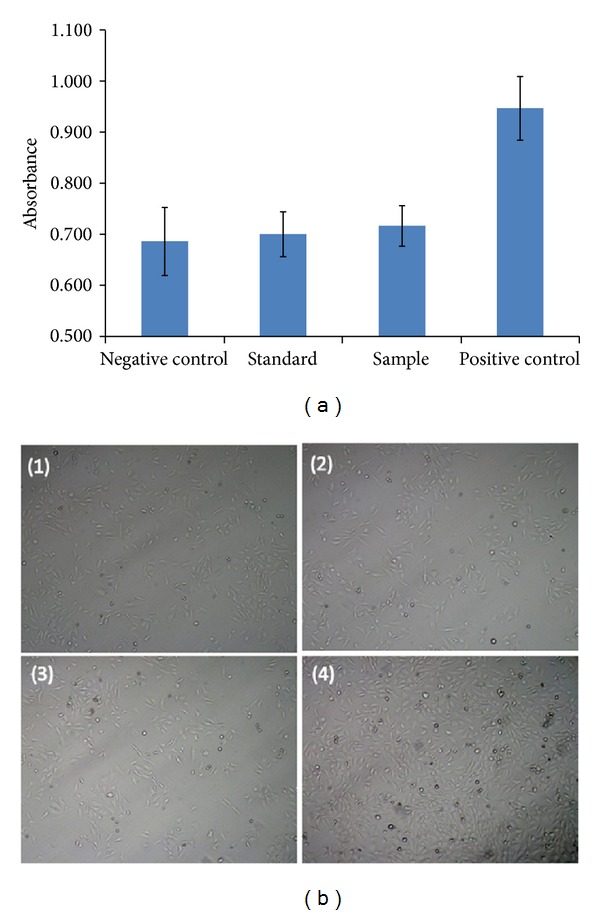
Cell proliferation assay performed on RF/6A cells to evaluate the biological activity of bevacizumab (a) absorbance produced by live cells, (b) images of live cells including (1) negative control (untreated cells), (2) standard (cells exposed to 100 ng/mL of VEGF and 0.25 mg/mL of bevacizumab), (3) sample (cells exposed to 100 ng/mL of VEGF and 0.25 mg/mL of released bevacizumab), and (4) positive control (cell exposed to 100 ng/mL VEGF). Results are described in mean ± SD, *n* = 6.

A cell proliferation assay was performed as described previously [29]. RF/6A cells proliferated rapidly in presence of VEGF (100 ng/mL, +ve control). However, proliferation was inhibited in absence of VEGF indicating sensitivity of endothelial cells towards growth factor, particularly, VEGF (Figure 18). Native bevacizumab (standard group) strongly inhibited VEGF-induced cell proliferation at a concentration of 0.25 mg/mL. A similar level of inhibition was also observed when released (from NPs) bevacizumab (test/sample group) was exposed for 24 h to VEGF treated cells. Moreover, inhibitory effects of released and native bevacizumab were not significantly different than −ve control.

### 3.8. Cell Migration Assay

**Figure 19:**
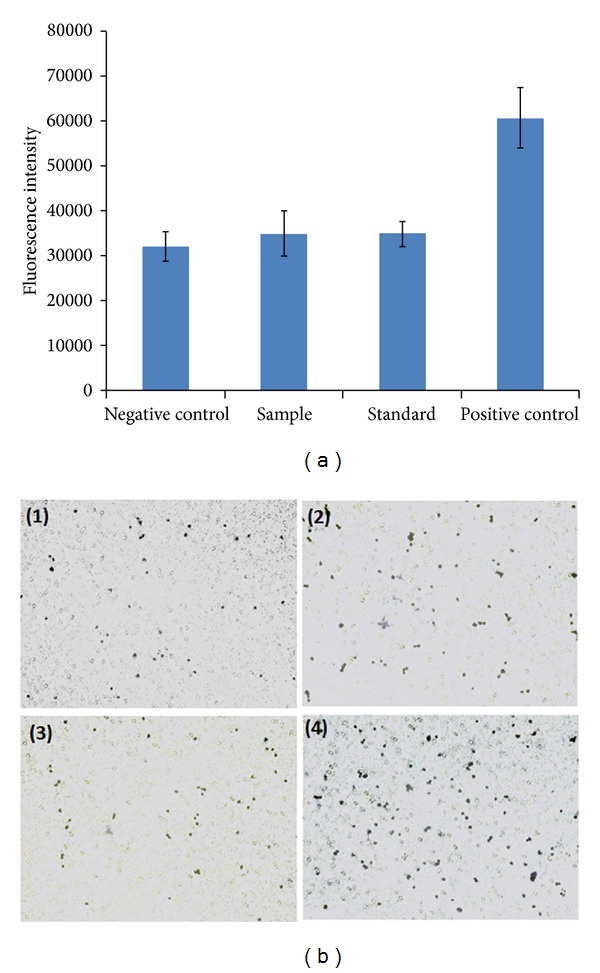
Cell migration assay performed on RF/6A cells to evaluate the biological activity of bevacizumab (a) fluorescence intensity indicating number of migrated cells, (b) images of migrated cells stained by methylene blue including (1) negative control (untreated cells), (2) standard (cells exposed to 100 ng/mL of VEGF and 0.25 mg/mL of bevacizumab), (3) sample (cells exposed to 100 ng/mL of VEGF and 0.25 mg/mL of released bevacizumab), and (4) positive control (cell exposed to 100 ng/mL VEGF). Results are described in mean ± SD, *n* = 6.

VEGF carries chemoattractant property which stimulates migration of RF/6A cells across a porous membrane towards a VEGF stimulus. Biological activity of released bevacizumab was further evaluated by VEGF-induced cell migration assay. As described in Figure 19, a test/sample group (released bevacizumab) exhibited significant inhibition of cell migration across transwell membrane compared to VEGF treated group (+ve control). Results indicated that this inhibitory effect was not significantly different than −ve control or a standard group (cells treated with native bevacizumab). For visual evidence, migrated cells were stained with methylene blue and images were prepared.  Figure 19(b) also supports the results described earlier.

Cell proliferation and migration assays clearly suggest that bevacizumab activity is retained during NP preparation and release. Previous reports suggest that PLA and/or PGA based copolymers produce large molar mass of lactic acid and/or glycolic acid [16, 17]. These degradation products stimulate hydrolytic degradation of protein therapeutics. Retention of protein stability (IgG and bevacizumab) in PB NPs can be attributed to lower molar mass of PLA or PGA blocks which produce very low amounts of lactic acid or glycolic acid, thereby eliminating or reducing the possibilities of protein degradation.

## 4. Conclusion

This research article discusses synthesis and characterization of novel PB copolymers for the preparation of NPs and thermosensitive gel. In order to eliminate burst release phase, a novel composite formulation comprised of protein-loaded PB NPs suspended in PB thermosensitive gel was successfully formulated and evaluated. FITC-BSA and IgG-encapsulated NPs suspended in thermosensitive gel demonstrated continuous zero-order release avoiding any possibility of dose-dependent toxicity. Bevacizumab-loaded NPs without gel demonstrated the same patterns of burst release and sustained release as those of IgG-embedded NPs. Retention of functional activity of bevacizumab by* in vitro* cell proliferation and migration suggests that this approach can act as a platform for the ocular delivery of therapeutic macromolecules. Such a system can minimize side effects associated with frequent intravitreal injections.

## Figures and Tables

**Table 1:** Characterization of polymers.

Code	Structure	Total Mn^a^ (theoretical)	Total Mn^b^ (calculated)	Total Mn^c^ (calculated)	Mw^c^ (GPC)	PDI^c^
PB-A	PGA_300_-PCL_7500_-PEG_1000_-PCL_7500_-PGA_300_	16600	15705	13781	18643	1.35
PB-B	PLA_3450_-PCL_5700_-PEG_4000_-PCL_5700_-PLA_3450_	22300	21034	19156	26978	1.41
PB-C	PEG_550_-PCL_825_-PLA_550_-PCL_825_-PEG_550_	3300	2910	4227	6044	1.43

a: theoretical weight of polymer.

b: molecular weight based on H-NMR.

c: molecular weight and PDI based on GPC.

**Table 2:** Characterization of FITC-BSA and IgG-loaded NPs.

Proteins	Copolymers	Entrapment efficiency (%)	Loading (%)	Particle size	Polydispersity
FITC-BSA	PB-A	34.98 ± 3.73	5.02 ± 0.39	351.5 ± 30.7	0.286
	PB-B	69.54 ± 6.23	5.42 ± 0.46	323.1 ± 24.7	0.280

IgG	PB-A	40.51 ± 3.46	6.31 ± 0.27	367.9 ± 11.9	0.305
	PB-B	70.06 ± 4.12	6.07 ± 0.31	346.4 ± 3.9	0.273

Bevacizumab	PB-B	67.34 ± 3.28	6.12 ± 0.22	337.8 ± 18.4	0.246

Results are described in mean ± SD, *n* = 3.

**Table 3:** Coefficient of determination (*R* ^2^) for various kinetic models for *in vitro* release of FITC-BSA, IgG, and bevacizumab.

Block copolymers	Korsmeyer-Peppas		Higuchi	Hixson-Crowell	First-order	Zero-order	Best fit model
	*R* ^2^	*n*	*R* ^2^	*R* ^2^	*R* ^2^	*R* ^2^	
FITC-BSA PB-B NPs	0.989	0.327	0.945	0.905	0.984	0.783	Korsmeyer-Peppas
IgG NPs PB-B NPs	0.982	0.334	0.979	0.979	0.959	0.898	Korsmeyer-Peppas
Bevacizumab PB-B NPs	0.977	0.344	0.969	0.961	0.959	0.881	Korsmeyer-Peppas
FITC-BSA PB-B NPs suspended in gel	0.992	0.549	0.978	0.959	0.982	0.851	Korsmeyer-Peppas
IgG PB-B NPs suspended in gel	0.997	0.818	0.971	0.996	0.987	0.994	Korsmeyer-Peppas
